# Validation of a scale to assess adherence to oral chemotherapy based on the experiences of patients and healthcare professionals (EXPAD-ANEO)

**DOI:** 10.3389/fphar.2023.1113898

**Published:** 2023-03-09

**Authors:** Amparo Talens, Elsa LÓpez-Pintor, Mercedes Guilabert, Natalia Cantó-Sancho, María Teresa Aznar, Blanca Lumbreras

**Affiliations:** ^1^ Pharmacy Department, Elda General University Hospital, Elda, Spain; ^2^ Department of Engineering, Area of Pharmacy and Pharmaceutical Technology, Miguel Hernandez University, Alicante, Spain; ^3^ Center for Biomedical Research in Epidemiology and Public Health Network (CIBERESP), Madrid, Spain; ^4^ Department of Health Psychology, Miguel Hernandez University, Elche, Spain; ^5^ Department of Optics, Pharmacology and Anatomy University of Alicante, Alicante, Spain; ^6^ Pharmacy Department, Hospital Universitario de San Juan de Alicante, Alicante, Spain; ^7^ Department of Public Health, History of Science and Gynecology, Miguel Hernandez University, Alicante, Spain

**Keywords:** oral antineoplastic, patient-reported experiences, validated scale, medication adherence, healthcare professional (HCP)

## Abstract

**Background:** Lack of adherence to Oral antineoplastic agents (OAAs) treatment has important clinical, social and economic consequences.

**Objective:** To develop and validate a novel instrument for assessing adherence to OAAs, based on the reported experiences of people with cancer in relation to their treatment and the opinions of the healthcare professionals who care for them.

**Methods:** We performed a multicenter validation study of a scale designed to assess adherence to OAAs. First, a steering committee developed the items for an initial scale, based on the results of a qualitative study that evaluated patients’ and professionals’ experiences with this treatment. We then assessed the validity and reliability of the initial scale in a sample of 268 outpatients with cancer who received their OAAs from four Spanish hospitals.

**Results:** The mean age of the sample of 268 outpatients was 64.1 (standard deviation [SD] 12.4) years, and 47% of participants were women. With the results of this analysis, we developed the EXPAD-ANEO scale, which has 2 factors, one for beliefs and expectations and another for behavior. Both factors explain 52% of the explained common variance. Good reliability was obtained, with a McDonald’s omega of 0.7 for the first factor and 0.6 for the second factor. The fit indices were optimal (Root Mean Square Error of Approximation = 0.02, Comparative Fit Index = 0.99, Tucker Lexis Index = 0.99 and Standardized Root Mean Squared Residual = 0.03), which verifies the appropriateness of the items to the model. We measured EXPAD-ANEO criterion validity against pill count, obtaining a specificity of 80%. We measured convergent validity with the Morisky-Green test and found a significant association (*p* < 0.001). We measured divergent validity with questions on health literacy from the 16-item European Health Literacy Survey and found no correlation (*p* = 0.153).

**Conclusion:** EXPAD-ANEO is the first validated instrument for evaluating patients’ experiences with and adherence to OAAs, providing valuable information that can help health professionals to establish individual strategies or collective programs for improving therapeutic results and reducing healthcare costs.

## 1 Introduction

People with cancer are increasingly prescribed oral antineoplastic agents (OAAs), which have several advantages over intravenous chemotherapy (Borner et al., 2001; McCue et al., 2014). However, the patients themselves, or their carers, are responsible for administering this treatment as prescribed, and lack of adherence can have serious clinical, social and economic consequences (McCue et al., 2014). Non-adherence compromises therapeutic efficacy, reducing the health outcomes and quality of life of people with cancer, while increasing associated health costs (Noens et al., 2009; Daouphars et al., 2013).

There is substantial heterogeneity between the instruments currently used to measure adherence to OAAs and the results of different studies are difficult to compare. A systematic review by Greer and colleagues indicated that adherence to oral treatment in people with cancer was between 46% and 100% (Greer et al., 2016). Two studies conducted in Spain reported adherence rates ranging from 72% to 79% (Olivera-Fernandez et al., 2014; Fernández-Ribeiro et al., 2017). Most tools currently in use are validated for other chronic diseases (Silveira et al., 2021). In people with cancer, one of the most frequently used tools is the Morisky-Green test (Huang et al., 2016). There have been initiatives to develop scales for measuring adherence to OAAs, usually in people with specific types of cancer (Bagcivan and Akbayrak, 2015; Gambalunga et al., 2022), or in people using specific drugs, such as imatinib (Daouphars et al., 2013). The definition of adherence among OAA users varies depending on the method of measurement, and there is no consensus in the literature, although any result other than 100% represents an opportunity for improvement.

The recommended option for determining adherence to treatment is to combine different methods, including pill count and personalized interviews with validated instruments (Morisky et al., 2008). This approach has been shown to provide a good approximation of real adherence (Solán, Sorli Redó and García, 2007). However, there are few initiatives in the literature to validate adherence scales or questionnaires in OAA users (Claros et al., 2019; Huang et al., 2016). Some studies have included very small patient samples (Peng and Wu, 2020), and the results of others have shown limited psychometric properties (Silveira et al., 2021). Furthermore, there are currently no references in the Spanish population, although research has shown that social and cultural characteristics may directly influence the barriers to and facilitators of adherence to OAAs (Irwin and Johnson, 2015). Understanding and taking into account how medication affects people’s daily lives is therefore crucial when designing specific tools for people using these drugs.

A previous qualitative study by our research team identified and evaluated the main barriers to and difficulties with correct use of OAAs as perceived by users, and the priorities of professionals who care for them (Talens et al., 2021). Using the results of this previous study as a starting point, we aimed to develop and validate a scale to measure adherence to OAAs based on the pharmacotherapeutic experience of people with cancer and the perspectives of the relevant healthcare professionals.

## 2 Methods

### 2.1 Study design

We conducted a multicenter validation study of a scale designed to measure OAA adherence in outpatients with cancer, based on their pharmacotherapeutic experience and the perspectives of the professionals who care for them. We named our instrument the EXPAD-ANEO scale after the Spanish abbreviation of EXPeriencia con y ADherencia a AntiNEoplasicos Orales (experience with and adherence to oral antineoplastic agents).

**FIGURE 1:**
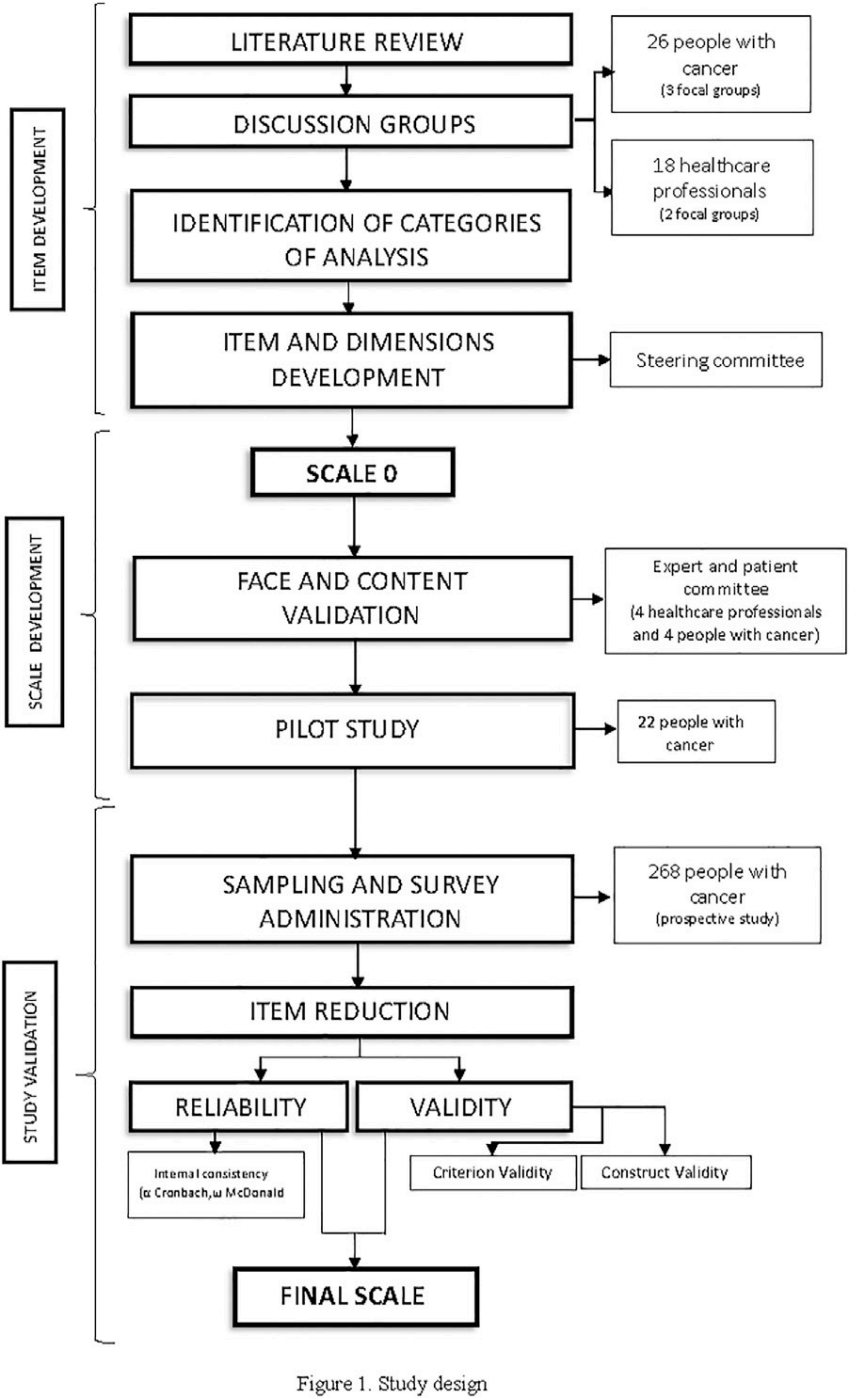
Study design.

The design process involved three stages, as described by Boateng et al. (2018) and colleagues: item development, scale development and scale evaluation (Figure 1). We also considered the existing standards and guidelines for validation practices (Chan, 2014), and the COSMIN recommendations (Mokkink et al., 2010).

### 2.2 Scope of the study

We included a sample of people with cancer who collected their oral antineoplastic treatment from the hospital pharmacies of four hospitals in the Valencian Community (Spain). The coordinating center was Elda General University Hospital, which has 548 beds and serves a geographically dispersed population of 189,629 inhabitants. The largest site—Alicante General University Hospital—is the tertiary care hospital of the province, with 841 beds and a catchment population of 280,535 inhabitants; while San Juan General University Hospital has 407 beds and serves 225,153 inhabitants; and Elche General University Hospital has 448 beds and serves 169,599 inhabitants.

### 2.3 Developing items to include in the scale

We organized a steering committee of six healthcare professions (four pharmacists, one physician and one psychologist), whose role was to construct the initial scale and take the necessary decisions throughout the validation process to define a reliable and valid final version. The working method we chose to approve the different versions of the scale was consensus conference (Martín-Delgado et al., 2013), so that the team could develop and reformulate the items after the various rounds of analysis and discussion.

In an initial phase based on qualitative research methods and scientific literature review, the committee analyzed the categorized discourse on the experiences and perspectives of people with cancer and healthcare professionals to identify the main dimensions explored in the qualitative study.

This previous qualitative study aimed to assess the medication experience in cancer patients undergoing ANEO treatment. The results of this study described what the patients perceived as barriers and facilitators to adherence and compared them with the healthcare professionals’ perspectives. Eight dimensions were initially identified: 1) treatment experiences, 2) polymedication, 3) beliefs regarding medication, 4) need for treatment and expectations about effectiveness 5) information and sources relating to the treatment 6) medication errors and forgetting to take medication and how to prevent this; 7) adverse effects and consequences of the treatment with ANEO; and 8) social, family and professional support. The main results of this study showed that the presence of adverse effects, lack of information about treatment, beliefs, needs and expectations regarding medications, social and family support, and the relationship with the health professionals were the most impactful aspects in the medical experience of patients in treatment with ANEO.

With this information, the steering committee established a proposal of dimensions for the initial scale through an iterative process involving deductive methods based on the literature review and inductive methods based on the discussions with patients and professionals.

### 2.4 Designing the scale

For the responses to the items, the steering committee designed a five-point Likert scale (1 = never, 2 = almost never, 3 = sometimes, 4 = almost always, 5 = always), to ensure efficiency in the subsequent validation. The committee also decided to vary the direction of the responses so that respondents would not detect a pattern (i.e., answering ‘never’ could represent high or low adherence, depending on the question).

We also formed an expert panel to evaluate content validity of the dimensions and items of the initial scale (ensuring there were no redundant or missing questions) and face validity (checking whether the questions were clear and easy to answer, whether the scale was adequate and whether respondents would understand the structure and response scale). For this panel, we recruited eight people through purposive sampling (Otzen and Manterola, 2017): four were healthcare professionals (two medical oncologists and two pharmacists) with at least 5 years of experience in cancer management, treatment and research; and four were people with cancer who had been on OAAs for more than 3 months. The results of this qualitative evaluation prompted changes in the reactive items of the scale.

Subsequently, we carried out a pilot study in a group of 22 people with cancer to identify any comprehension difficulties and to evaluate the functioning of our scale. We consecutively included adults (aged 18 years or older) with a cancer diagnosis when they attended or contacted one of the participating hospital pharmacies to collect or request their treatment. Eligible treatments belonged to the subgroups L01 (Antineoplastic agents) and L02 (Endocrine therapy) and L04 (Immunosuppressants) of the Antineoplastic and immunomodulating agents’ group (L) of the Anatomical Therapeutic Chemical (ATC) Classification System. We included only people who had been on the treatment for more than 1 month. We excluded people with communication difficulties or who refused to participate in the study. We measured the time taken to complete the questions and we analyzed whether the items addressed participants’ full experience. After completing the scale, participants were asked whether they had difficulty understanding any of the questions and whether they considered the response scale to be adequate.

### 2.5 Validation of the scale: Tests of reliability and validity

We performed a prospective evaluation in cancer outpatients on OAAs recruited between March and November 2021, to collect data that would help us to determine the validity and reliability of the EXPAD-ANEO scale.

To ensure homogeneous data collection in the four participating hospitals, we provided specific training to the pharmacists who would collect the data.

#### 2.5.1 Selection criteria

People eligible for study inclusion were aged 18 or over, had a cancer diagnosis, and had been receiving OAAs (ATC code L01, L02 or L04) for at least 3 months. We excluded people with communication or comprehension difficulties.

#### 2.5.2 Sample size calculation

We calculated a sample size of 268 patients from an infinite population, assuming 78% adherence in the Spanish population (Fernández-Ribeiro et al., 2017), and applying a confidence level of 95% and precision of 5%. The necessary sample size to validate an instrument varies according to the number of items (10 respondents per item (Boateng et al., 2018)) or dimensions, but the minimum recommended number to ensure stable and generalizable results is 175–200 participants (Hair et al., 2009).

#### 2.5.3 Recruitment

The team of pharmacists in each participating hospital consecutively recruited eligible people until reaching the predefined sample size. When a person receiving OAAs contacted the hospital pharmacy to collect their medication, the pharmacist checked whether they met the eligibility criteria and, if so, invited them to participate in the study. Prior to inclusion, the potential participant received information on the study objectives and an informed consent form. If they agreed to the conditions and gave their informed consent, a telephone interview was scheduled. The increased use of telepharmacy in Spanish hospitals since the COVID-19 pandemic facilitated this process. The pharmacists were responsible for conducting the interview and evaluating therapeutic adherence.

#### 2.5.4 Study variables

We collected sociodemographic and clinical variables of participants, such as age, sex, educational attainment (no schooling/primary education/secondary education/tertiary education), ECOG score (0–4), living situation (alone/with family/institutionalized), diagnosis, treatment objectives (adjuvant/palliative), treatment duration, line of treatment, adverse effects, and health literacy. We measured health literacy on a small scale of six questions selected by the steering committee from the 16-Item European Health Literacy Survey (HLS-EU-Q16) (Nolasco et al., 2020), to measure the association with adherence.

We evaluated the dependent variable, adherence to OAAs, using the new EXPAD-ANEO scale, the hospital pharmacy dispensing records, the Morisky-Green test and the pill count method, which we used as the benchmark.

All variables were obtained from the hospital electronic medical records of the Valencian Community (Orion Clinic and Abucasis) and the telephone interviews.

#### 2.5.5 Validity and reliability

Table 1 lists all the indexes and statistics calculated to assess the reliability and validity of the instrument, with the correspondent cut-off values to be applied to each methodology.

• Item reduction and dimensionality analysis: to check the relationship between items, we applied Bartlett’s test of sphericity. We carried out an exploratory factor analysis using the maximum likelihood extraction method, and a principal component analysis with an oblique rotation method. We also performed an optimal implementation of parallel analysis to determine the fit of the items to the model and whether any of them should be eliminated based on the measure of sampling adequacy (MSA) index. MSA values below 0.50 suggest that the item does not measure the same domain as the rest of the items and should therefore be dropped (Lorenzo-Seva and Ferrando, 2021). We did not consider factor loadings of less than 0.3. Subsequently, we carried out a confirmatory factor analysis to evaluate the fit of the model obtained in the exploratory factor analysis. To assess the fit of the model, we included the following robust goodness of fit statistics: 1) Root Mean Square Error of Approximation (RMSEA), considering as admissible adjustment values of 0.06 or less; 2) Comparative Fit Index (CFI), where values above 0.95 would be adequate; 3) Tucker Lexis Index (TLI), where values above 0.97 indicate a good model fit; and 4) Standardized Root Mean Squared Residual (SRMR), where values below 0.10 would be adequate (Cangur and Ercan, 2015). In addition, we calculated the total percentage of variance explained, and whether the set of items that make up the instrument had a given unidimensional or multidimensional structure.
• Reliability: degree to which an instrument is able to measure without errors, i.e., to measure accurately and consistently over time. We used McDonald omega coefficient to determine the internal consistency of the items and how they relate to each other, both for the global scale and for the single item. McDonald’s omega coefficient is intended to replace Cronbach’s alpha given that the instrument has a certain multidimensional structure. The instrument demonstrates an acceptable reliability when the omega coefficients is greater than 0.6 (Dunn et al., 2014), (Nájera Catalán, 2019).
• Criterion validity: to assess the criterion validity of the questionnaire in the absence of a gold standard for measuring adherence, we used pill count as a surrogate, as it is one of the most commonly used methods in daily practice. Participants with a pill count of 90% or more were considered adherent to treatment (Daouphars et al.). We determined the cut-off point with the highest specificity for classifying participants as adherent or non-adherent.
• Construct validity based on known groups: to assess the construct validity of the questionnaire, we followed the strategy of comparing two groups established according to the Morisky-Green test, as it is the most widely used in people with cancer (convergent validity) (Morisky et al., 1986). For this study, we used the four-question version that classifies respondents as adherent or non-adherent; it is validated for different chronic pathologies and is widely used in research. To test divergent validity, we compared the EXPAD-ANEO scores with the health literacy scores. We calculated the chi-squared (X^2^) statistic and correlation coefficient to evaluate potential heterogeneity between groups.

**TABLE 1:** Indexes, statistics, and coefficients used to assess the reliability and validity of the instrument.

Psychometric properties		Name of index, statistic, or coefficient	Abbreviation	Adequate value
**Reliability**	Internal consistency	McDonald omega coefficient	ω	>0.6
**Validity**	Redundancy between items	Bartlett’s test of sphericity	—	*p*-value <0.001
	Adequacy of the items to the model	Measure of Sampling Adequacy	MSA	>0.5
	Model’s fit	Root Mean Square Error of Approximation	RMSEA	≤0.06
		Comparative Fit Index	CFI	>0.95
		Tucker Lexis Index	TLI	>0.97
		Standardized Root Mean Squared Residual	SRMR	<0.10

For the statistical analyses, we used SPSS version 28 and Jamovi 1.6.23.

### 2.6 Ethical considerations

This study received a favorable opinion from the Institutional Review Board of Elda General Hospital on 14 April 2020 (PI 2020/12), and subsequent amendments in March 2021 were also approved.

It is registered in ClinicalTrials.gov under the Identifier NCT04550533 (clinicaltrials.gov/ct2/show/NCT04550533).

## 3 Results

### 3.1 Development of the items

With reference to the eight categories identified in the qualitative study (Talens et al., 2021), and after reviewing the dimensions of other instruments, the steering committee created the following five exploratory dimensions: Experience with the treatment, Beliefs and expectations, Sources of information and support, Errors, Forgetfulness and polypharmacy, and Side effects. Subsequently, the steering committee generated several items for each dimension (30 in total), based on the scientific literature and the productivity measures of ideas provided, spontaneity and consistency in the group discussions with patients and professionals.

In different meetings, after the expert analysis in the consensus conference, the committee eliminated six items that it deemed redundant, and four items with responses that could not be adapted to the Likert scale (although we collected these four variables in the patient interviews because they provided information on participants’ sociodemographic characteristics and health literacy). In this way, we generated the first version of the instrument, which had four main dimensions. The committee initially grouped the eight dimensions described in the qualitative analysis into four categories according to analogy and similarity in the items included in each dimension. Thus, the dimension of beliefs regarding medication was combined with the dimension of need for treatment and expectations of effectiveness. In the same way, the dimension of information about treatment was combined with social, family and professional support, and these aspects were considered as facilitators of adherence; the dimension of polymedication was combined with the category of medication errors, failures and forgetting to take medication as well as the dimension of adverse effects into a single dimension because all of them are problems related to medication. The four resulting categories were 1) ANEO experiences, 2) Beliefs and expectations, 3) Information and support, 4) Problems related to ANEO.

### 3.2 Design of the scale

#### 3.2.1 Face and content validity

Eight participants (four outpatients and four professionals) evaluated the face and content validity of the instrument. The average age of the outpatients was 59.2 (standard deviation [SD] 7.5) years, and half were women. Two professionals were oncologists and two were pharmacists. The average age of the professionals was 46 (SD 9) years, and one was a woman. Half of respondents thought the scale was understandable, while the rest suggested removing the abbreviations and changing the wording of items 2, 5, 7 and 17. All respondents agreed that the items were relevant and all understood the response scale, although two respondents said they would prefer a simpler scale, with two response choices. Regarding the length of the questionnaire, three respondents found it too long, two though it was too short and the remaining three considered it suitable. Using the respondents’ comments, we reformulated four items, spelled out the abbreviations and changed the order of the questions. Although two participants preferred dichotomous questions, we maintained the five-point Likert scale after consulting with experts, who agreed on the efficiency of rating scales.

#### 3.2.2 Pilot study

The pilot study included 22 of 25 outpatients who we had invited to participate. The average age of the group was 68.7 (SD 7.6) years, and 41% were women. Most participants (86%) lived with their family, 50% had no schooling and 73% were retired. Thirteen participants (59%) were treated in the oncology department. The median time from diagnosis was 35 months (interquartile range (IQR) 87 months), and the most common diagnoses were prostate cancer (18%) and multiple myeloma (18%). Nearly two-thirds of participants (64%) used multiple medications, and the most common anticancer drugs were ibrutinib (18%), capecitabine (18%) and abiraterone (14%). Following the pilot study, we reformulated eight questions based on the participants’ recommendations.

### 3.3 Validation of the scale

#### 3.3.1 Included patients

The pharmacists from the four hospitals invited a total of 350 people to participate, of whom 24 (8%) refused, 35 (11%) were unable to respond to the questions or had communication difficulties, and 23 (9%) were ineligible as they had been on the treatment for less than 3 months.

We included 268 patients (77%). Each had a telephone interview with a pharmacist, lasting approximately 15 min. The aim of this initial contact was to build trust between the participants and the investigators. To ensure accuracy in the responses, we limited the recall period to 1 month. The mean age of the participants was 64.1 (SD 12.4) years (range 25–91 years), and 47% were women. Most participants (88%) lived with their family, 18% had no schooling and 57% were retired. Sixty-one per cent of participants were managed in oncology departments and 39% in hematology departments. The median duration of OAA treatment was 12 months (range 5–29 months). The most common diagnoses were multiple myeloma (16%), breast cancer (14%), chronic lymphocytic leukemia (10%) and prostate cancer (10%). In 83% of participants, the goal of treatment was palliative. OAAs constituted the first treatment for 68%. Most participants (59%) were receiving more than five drugs, and the most common anticancer agents were lenalidomide (15%), ibrutinib (11%), capecitabine (9%) and abiraterone (7%).

#### 3.3.2 Validation of the scale

We collected data prospectively using the 20-item scale (four dimensions) we had created through the process described above (Supplementary Table S1).

• Item reduction and dimensionality analysis: after a descriptive analysis of all variables, we observed that Q14 (Have you stopped taking your medication at any point on the recommendation of someone in a similar situation?) was a constant, so we removed it from the scale. In all other questions except Q3, we identified an important floor/ceiling effect (respondents tended to select ‘never’ or ‘always’); we therefore decided to dichotomize the responses (Yes/No). All items had values in both categories. The *p*-value of Bartlett’s test was below 0.001, indicating a relationship between the items. After carrying out the exploratory factor analysis and the principal component analysis, we found that 12 questions had an MSA index below 0.5, so we eliminated them from the scale. This left seven items in the final scale (Q4, Q5, Q15, Q16, Q17, Q18 and Q19). The factor analysis extracted two factors accounting for 52% of the explained common variance. The factor loading values ranged from 0.34 (Q5) to 0.88 (Q4) as shown in Table 2. We verified appropriateness using the robust goodness of fit statistics, obtaining the following results: RMSEA = 0.02, CFI = 0.99, TLI = 0.99 and SRMR = 0.03.
• Reliability: As this is a two-dimensional instrument (2 factors), we calculated the omega coefficients for each factor separately and for each item if it were removed. Table 3 presents the result of this analysis. McDonald’s omega coefficient showed a reliability of 0.7 for factor 1 and 0.6 for factor 2. Considering the omega coefficient, the range of correlations of each item with the total score was 0.4 (Q4 and Q17) to 0.8 (Q5).
• Criterion validity: Regarding criterion validity, we found that a cut-off of 1 point would optimize the specificity of the questionnaire at 80%. This means that a person scoring 1 point or more on the questionnaire was considered non-adherent to treatment.
• Construct validity based on known groups: finally, when we assessed convergent validity against the Morisky-Green test, the X^2^ test showed a significant association (P < 0.001). Regarding divergent validity, we first verified the normality of the two variables (score on the questionnaire we are validating and score on the literacy questionnaire); as the variables were not normally distributed, we used Spearman’s correlation coefficient rather than Pearson’s correlation coefficient. We found a correlation coefficient of 0.087 (P = 0.153), indicating no correlation between the two questionnaires.

**TABLE 2:** Factor analysis.

Variable	FLV	MSA
Factor 1		
Q4. Do you sometimes stop taking the antineoplastic because you think it is useless?	0.88	0.55
Q5. Do you sometimes think that another intravenous/transplant drug would produce better results than the current oral drug?	0.34	0.64
Q19. Do you sometimes stop taking the drug when you feel well for fear of feeling ill?	0.71	0.55
Factor 2		
Q15. Do you sometimes miss a dose of your chemotherapy when you feel sick?	0.55	0.59
Q16. Do you sometimes stop taking the chemotherapy without consulting your doctor because it drains your energy and makes you tired?	0.37	0.70
Q17. Do you sometimes miss a dose of your chemotherapy for fear of reactions like vomiting, cramps, diarrhea or skin problems?	0.77	0.59
Q18. Do you sometimes stop taking your chemotherapy because you are worried it will affect your work or social life?	0.37	0.67
Total		0.59

FLV: factor loading value; MSA: measure of sampling adequacy.

**TABLE 3:** McDonald’s omega (ω) coefficients.

	Item reliability statistics (if item dropped)	Scale reliability statistics
Variable	ω	ω
Q4	0.4	0.7
Q5	0.8	
Q19	0.5	
Q15	0.5	0.6
Q16	0.6	
Q17	0.4	
Q18	0.6	
Total		0.6

After the validation procedure, the scale finally included 2 dimensions and 7 items.

### 3.4 Interpretation of the new adherence scale

Table 4 presents the results of the validation: the two-dimension, seven-item EXPAD-ANEO scale. The dimension related to beliefs and expectations finally includes three items. We grouped items that described patient’s attitudes affecting adherence in a new dimension denominated behaviour and attitudes. This new dimension includes four items, mainly those describing medication-related problems.

**TABLE 4:** EXPAD-ANEO scale.

Beliefs and expectations about treatment
Q4. Do you sometimes stop taking the antineoplastic because you think it is useless?
Q5. Do you sometimes think that another intravenous/transplant drug would produce better results than the current oral drug?
Q19. Do you sometimes stop taking the drug when you feel well for fear of feeling ill?
BEHAVIOR AND ATTITUDES
Q15. Do you sometimes miss a dose of your chemotherapy when you feel sick?
Q16. Do you sometimes stop taking the chemotherapy without consulting your doctor because it drains your energy and makes you tired?
Q17. Do you sometimes miss a dose of your chemotherapy for fear of reactions like vomiting, cramps, diarrhea or skin problems?
Q18. Do you sometimes stop taking your chemotherapy because you are worried it will affect your work or social life?

Due to the extreme responses (at both ends of the scale) obtained in the validation, the steering committee decided to reduce the response scale to two options.

Possible scores range from 0 to 7 points, with each affirmative answer adding 1 point. Because the instrument is highly specific, a respondent who gives only one affirmative answer is considered non-adherent. According to the results of the EXPAD-ANEO scale, 20% of people interviewed were non-adherent to their oral antineoplastic treatment. The mean of the scale for the whole population was 0.28 (SD 0.66), and scores ranged from 0 to 4. Among the non-adherent participants, the mean score was 1.41 (SD 0.77), and scores ranged from 1 to 4.

## 4 Discussion

By adopting a methodological approach that included qualitative research techniques, we were able to design a tool through orderly discussion, where people with cancer were at the center of the process from the outset and participated in the development, design and validation stages. To the best of our knowledge, this is the first study to incorporate the experiences and opinions of people with cancer and professionals in the development of a scale that measures adherence to OAAs. Most studies of this type are based on literature reviews and expert opinions only (Claros et al., 2019a). Peng and colleagues conducted a study with similar methods in a Chinese population, although their scale did not measure adherence specifically but rather self-management of oral chemotherapy, without considering other anticancer approaches such as hormonal or targeted therapy (Peng and Wu, 2020). One recent publication describes the development of the A-BET questionnaire, which involved a qualitative study in people with breast cancer receiving hormone therapy, and a subsequent validation stage; however, the sample size was very small (Gambalunga et al., 2022). One aspect that sets our study apart is the wide range of treatments included (all oral antineoplastic agents dispensed in public Spanish hospitals).

In addition, the validation process enabled us to select the most appropriate items for evaluating adherence through measurement of their psychometric properties. Face and content validity assessment is important to ensure the items are relevant and represent the construct they are intended to measure. In the literature, we found that the development of these types of tests commonly involves expert opinions (Lessa et al., 2015; Baudot et al., 2016) or pilot studies in people with the same characteristics (Urzua et al., 2010). In our study, the double evaluation (by the group of experts followed by the pilot study in people with cancer) conferred validity and coherence to our instrument. Bagcivan and colleagues used a more sophisticated model to determine the quantitative content validity index (Bagcivan and Akbayrak, 2015).

Compared with other scales, EXPAD-ANEO showed acceptable reliability, which we evaluated with McDonald’s omega coefficient because it is currently considered a more sensitive measure than the commonly used Cronbach’s alpha, and more appropriate for estimating reliability, particularly of multidimensional instruments including different scales of items and factor loads (Dunn et al., 2014; da Silveira and Jorge, 2002).

A systematic review by Claros and colleagues included six validation studies. Only two of the included studies showed acceptable validity and reliability for measuring adherence in people with cancer: the Adherence Determinants Questionnaire (ADQ) (Lessa et al., 2015) and the Oral Chemotherapy Adherence Scale (OCAS) (Bagcivan and Akbayrak, 2015).

In a more recent study, the validation of the Treatment Adherence Measure (TAM) (Silveira et al., 2021) in outpatients with multiple myeloma was unsatisfactory. That study reported very high adherence to treatment and a tendency to extreme responses, as in our study. Evaluation of other adherence measurement tools has shown a ceiling effect: more that 15% of responses to each question represent highest adherence on the scale (da Silva Carvalho et al., 2010).

The factor analyses in our study extracted the seven items that made up the final scale: Q4, Q5 and Q19 grouped in a factor related to beliefs about and expectations of the treatment; and Q15, Q16, Q17 and Q18 included in a factor referring mainly to behaviors of treatment use. Tests like Morisky-Green usually explore only the second factor. The first factor of our instrument distinguishes it from other adherence scales, and provides the opportunity to explore patients’ thoughts, beliefs and expectations, and how these variables correlate to more or less adherent behavior. In addition, we responded to the floor/ceiling effect identified during the validation process by dichotomizing the responses (Yes/No), as in other published studies (Silveira et al., 2021; da Silva Carvalho et al., 2010).

Criterion validity is important because it compares our results with the gold standard. In the absence of an objective validated measure for OAA adherence, we used pill count (the most widely used method in daily practice), considering a value over 90% representative of adherence (Daouphars et al.; Timmers et al., 2014). The 80% specificity enabled us to detect non-adherence in a population that is generally considered very adherent, with only one affirmative response.

We confirmed convergent validity with the Morisky-Green test and divergent validity with the health literacy questions selected by the steering committee, with statistical significance in both cases. The Morisky Green test is the most widely used questionnaire in clinical practice to assess adherence and is validated in different chronic diseases such as hypertension and diabetes (Morisky et al., 2008). However, it has not been validated in cancer patients, despite its use for this pathology (Signorelli et al., 2022), which represents a limitation for its use in clinical practice. In addition, the Morisky-Green test aims to assess patient attitudes, assuming that if patients’ behaviour is good, the patient is adherent. In contrast, the result of our EXPAD-ANEO research, not only considers a patient’s behaviour, but also explores the patient’s beliefs associated with adherence and the need for treatment, as well as what they considered to be barriers to adherence. EXPAD-ANEO is therefore, a scale designed to include the patients’ differential social and cultural characteristics, the patient’s opinion and perspective, as well as the healthcare professionals’ approach. Moreover, given that this scale has been validated specifically for patients with cancer who are undergoing treatment with oral antineoplastic agents, it is a reliable scale for using in clinical practice.

Possible scores on the EXPAD-ANEO scale range from 0 to 7; 80% of our participants scored 0 points and were considered adherent. Respondents who scored just one point on this highly specific scale (15% of our population) were considered non-adherent. The grouping of the scores towards the two extremes of the scale indicated a lack of discriminating capacity in a population that is considered highly adherent. However, any result other than 100% shows room for improvement and the opportunity to detect isolated cases of non-adherence and avoid therapeutic failure.

### 4.1 Implications for clinical practice

A population of people with cancer in a specific social, cultural and economic context, with access to universal healthcare, showed high levels of adherence (80%), comparable with data from other studies in the literature (Greer et al., 2016), but there is still room for improvement. The systematic use of a simple, valid, reliable and highly specific instrument for detecting non-adherence in this population could help healthcare professionals to establish individualized measures or collective strategies to improve adherence and health outcomes. By helping physicians and pharmacists to better understand the personal aspects that influence patients’ use of medication, a tool like this could contribute to improving healthcare quality (Olivera-Fernandez et al., 2014; Gatwood et al., 2017; Middendorff et al., 2018; Birand et al., 2019; Passey et al., 2021).

Furthermore, the success of treatments and strategies increases when patients are placed at the center of the system and are encouraged to participate in decision-making with healthcare professionals (González-Bueno et al., 2018). Today, it is crucial to consider patients’ perspectives when evaluating and improving healthcare services (Boateng et al., 2018). This approach redirects health systems towards person-centered care (González-Bueno et al., 2018). In general, improving the quality and safety of patient care involves evaluating patient-reported experience measures and patient-reported outcomes measures (Valderas and Alonso, 2008), as we have in this study, along with other more objective indicators, with the aim of orienting the system towards increasingly integrated and humanized care, in which patients can take decisions about their own health (Martin-Delgado et al., 2021).

#### 4.1.1 Strengths

The EXPAD-ANEO scale is the first tool developed in a Spanish population for measuring adherence to OAAs. We adopted an innovative approach to designing and validating the scale, evaluating adherence in relation to the experiences of the people using the treatment while also incorporating the opinions of healthcare professionals. Throughout the design and validation process, we followed current standards, recommendations and expert consensus (Mokkink et al., 2010; Chan, 2014; Boateng et al., 2018).

In addition, our study included four hospitals and a large sample of outpatients who used a wide range of oral antineoplastic agents. Similar studies in the literature have included few patients and limited medications (Claros et al., 2019a; Huang et al., 2016).

#### 4.1.2 Limitations

One of the main limitations of this study was that we had no gold standard against which to validate our instrument. For the criterion validity assessment, we used pill count as a surrogate, for two main reasons: first, it is the method most widely used by hospital pharmacists when evaluating adherence in clinical practice; and second, it is the available method that most closely reflects real adherence, since use of electronic devices is limited to research. Another possible limitation is the variability in data collection resulting from the multicentric nature of the study. We tried to reduce this effect by training the hospital pharmacists before data collection. In addition, as with all scales, there is a risk of overestimating adherence, though we minimized this risk in our study by comparing the results of the EXPAD-ANEO scale with other measures such as pill count.

For practical reasons, we were unable to retest our scale on the same large sample of outpatients; as a result, reliability was limited to internal consistency. We had to delay the study during the COVID-19 pandemic, and the pharmacists were unable to collect data in face-to-face interviews as planned because most patients received their medication at home. In view of the increased implementation of telepharmacy (remote pharmaceutical care) in the Spanish health system, we decided to conduct the interviews over the telephone. This may have led patients to consider the questions less carefully and give extreme responses on the five-point scale.

The qualitative study initially described eight different dimensions which were then grouped into four dimensions and finally, after the validation of the scale through the factor analysis, it was transformed into a two-dimension scale. Although the initial scale included eight categories with more potential information, the factor analysis process eliminated redundant information and converted the more specific thematic units into more general ones. Hence, the final scale was easier to use in practice.

Finally, although we had originally aimed to vary the direction of responses in the scale, all seven questions that remained after item reduction had the same response pattern (Yes = non-adherent, No = adherent).

### 4.2 Future research

EXPAD-ANEO constitutes a starting point for developing this type of practical and sensitive instrument to helps professionals predict adherence based on the experiences of people with different chronic pathologies, including cancer, or even specific cancers or cancer stages. These instruments should be integrated into clinical practice as part of the routine clinical interview so that physicians can propose specific interventions without delay when they detect a possible lack of adherence. The new scale is designed to be collected by health professionals and the Morisky-Green test is a self-reported questionnaire. Further research could be carried out to evaluate the characteristics of this new scale so that it can be applied in a self-reported format to facilitate its incorporation in clinical practice.

As the validation of an instrument is not a static process, future studies should evaluate the EXPAD-ANEO scale’s sensitivity to change, i.e., the ability of the scale to detect changes in adherence to oral medication after an intervention, for example, by the size of the effect.

## 5 Conclusion

EXPAD-ANEO scale is a novel instrument with acceptable validity and reliability for systematically evaluating adherence to OAAs. It can serve as a starting point for future studies. Researchers can use this scale to explore patients’ experiences and adherence to treatment. Healthcare professionals can easily integrate this simple and applicable tool into their care routine during clinical interviews. It can help them to explore, in an unbiased way, the beliefs and behaviors of people with cancer in relation to their medical treatment.

## Data Availability

The original contributions presented in the study are included in the article/Supplementary Materials, further inquiries can be directed to the corresponding author.
