# Supplementary material for: Validation of a scale to assess adherence to oral chemotherapy based on the experiences of patients and healthcare professionals (EXPAD-ANEO)
Source: Front Pharmacol. 2023 Mar 9;14:1113898. doi: 10.3389/fphar.2023.1113898 (PMC10033971; doi:10.3389/fphar.2023.1113898)
Supplement: Supplementary file 1 [file Table1.DOCX]

Supplementary material. Table 1 . Initial EXPAD-ANEO scale

Please indicate how often you encounter the following situations:

|  | **Never** | **Almost never** | **Sometimes** | **Almost always** | **Always** |
| --- | --- | --- | --- | --- | --- |
| 1. Between visits to the hospital pharmacy, are you missing pills? |  |  |  |  |  |
| 2. Do you feel that knowing your medication and understanding it helps you to take it correctly? |  |  |  |  |  |
| 3. Have you ever felt that you take too many medications? |  |  |  |  |  |
| 4. Do you sometimes stop taking the antineoplastic because you think it is useless? |  |  |  |  |  |
| 5. Do you sometimes think that another intravenous/transplant drug would produce better results than the current oral drug? |  |  |  |  |  |
| 6. Do you take the oral medication because you think that the beneficial effects outweigh the negative things? |  |  |  |  |  |
| 7. Have you stopped taking it because you found contradictions between the information given to you by different health professionals (e.g., the primary care physician and the specialist physician (oncologist/haematologist)? |  |  |  |  |  |
| 8. Have you made a mistake in the dose or frequency of oral medication because you are taking too many drugs in your treatment? |  |  |  |  |  |
| 9. Have you stopped taking the medication because you think you are taking too many drugs? |  |  |  |  |  |
| 10. ¿ Does your daily activity, at work or in your leisure and free time, ever cause you to forget to take your medication? |  |  |  |  |  |
| 11. Can a change in your daily activity (dinner, travel, going out...) make you forget or miss a dose of the medication? |  |  |  |  |  |
| 12. Do you take your oral medication as your health care professional told you to take it? |  |  |  |  |  |
| 13. Do you need a family member or friend to remind you to take your medication? |  |  |  |  |  |
| 14. Have you stopped taking your medication because someone else in a similar situation recommended you do so? |  |  |  |  |  |
| 15. Do you sometimes miss a dose of your chemotherapy when you feel sick? |  |  |  |  |  |
| 16. Do you sometimes stop taking the chemotherapy without consulting your doctor because it drains your energy and makes you tired? |  |  |  |  |  |
| 17. Do you sometimes miss a dose of your chemotherapy for fear of reactions like vomiting, cramps, diarrhoea or skin problems? |  |  |  |  |  |
| 18. Do you sometimes stop taking your chemotherapy because you are worried it will affect your work or social life? |  |  |  |  |  |
| 19. Do you sometimes stop taking the drug when you feel well for fear of feeling ill? |  |  |  |  |  |
| 20. Have you ever felt sick with the oral MEDICATION and needed your doctor to prescribe a new course of treatment? |  |  |  |  |  |
